# Occurrence and Genetic Diversity of *Babesia caballi* and *Theileria equi* in Chilean Thoroughbred Racing Horses

**DOI:** 10.3390/pathogens10060714

**Published:** 2021-06-07

**Authors:** Reinaldo Torres, Claudio Hurtado, Sandra Pérez-Macchi, Pedro Bittencourt, Carla Freschi, Victoria Valente Califre de Mello, Rosangela Zacarias Machado, Marcos Rogério André, Ananda Müller

**Affiliations:** 1 Instituto de Ciencias Clinicas Veterinarias, Facultad de Ciencias Veterinarias, Universidad Austral de Chile, Valdivia 5090000, Chile; r.torres.celis@gmail.com (R.T.); churtadop@gmail.com (C.H.); 2 Departamento de Patologia Clinica Veterinaria, Facultad de Ciencias Veterinarias, Universidad Nacional de Asunción, San Lorenzo 1114, Paraguay; stperez@vet.una.py; 3 Biomedical Sciences Department, Ross University School of Veterinary Medicine, Basseterre, Saint Kitts and Nevis; pbittencourt@rossvet.edu.kn; 4 IMUNODOT Diagnostico, Jaboticabal 14887-042, SP, Brazil; crfreschi@gmail.com; 5 Departamento de Patologia, Reproducao e Saude Unica, Faculdade de Ciencias Agrarias e Veterinarias, Universidade Estadual Paulista, (FCAV/UNESP), Jaboticabal 14884-900, SP, Brazil; vick_vvc@hotmail.com (V.V.C.d.M.); rzacariasmachado@gmail.com (R.Z.M.); mr.andre@unesp.br (M.R.A.)

**Keywords:** equine piroplasmosis, babesiosis, theileriosis, ELISA, nested PCR

## Abstract

This study aimed to serologically and molecularly survey *Babesia caballi* and *Theileria equi* in thoroughbred horses from racecourses in Chile. Additionally, the genetic diversity of the positive samples was assessed. A total of 286 thoroughbred horses from the Santiago and Valparaíso racecourses had their serum samples submitted to an ELISA for *B. caballi* and *T. equi*, and 457 samples (from the Santiago, Valparaíso, and Concepción racecourses) were tested with nested PCRs for the *B. caballi* 48 KDa rhoptry protein (RAP-1) and *T. equi* 18S rRNA genes. Selected RAP-1 and 18S positive products were sequenced to perform phylogenetic and haplotype analyses. An overall seroprevalence of 35.6% was observed for these Chilean racecourses: 23.7% for *T. equi,* 8.4% for *B. caballi*, and 3.5% for both agents. Overall, a 53.6% occurrence by nPCR was detected for the three Chilean racecourses: 44.2% for *T. equi*, 5.4% for *B. caballi*, and 3.9% for both agents. Phylogenetic analysis of *T. equi* and *B. caballi* showed genetic proximity with sequences previously detected in other countries. Haplotype analysis revealed a low diversity among the Chilean sequences, which may have originated from those reported in Brazil, Israel, or Cuba. *Babesia caballi* and *T. equi* were detected for the first time in Chilean thoroughbred horses.

## 1. Introduction

Equine babesiosis and theileriosis, together known as equine piroplasmosis (EP), are two tick-borne diseases caused by the protozoans *Babesia caballi* and *Theileria equi* (Apicomplexa: Piroplasmida) [1], which belong to the Babesiidae and Theileriidae families, respectively [2]. EP has high morbidity and mortality rates and is, therefore, a serious concern in the equine industry [3]. *Babesia caballi* and *T. equi* are transmitted by ticks (biological vectors), and frequently co-infect horses [4,5,6,7]. Although transmission via the placenta from pregnant mares to fetuses has been described, this route is of little epidemiological significance for EP, and is considered rare, especially for *B. caballi* [8,9,10,11]. Additionally, *B. caballi* and *T. equi* can be transmitted mechanically or iatrogenically via contaminated needles and syringes, blood transfusions, and surgical instruments [9]. 

Both *B. caballi* and *T. equi* infections can be subclinical to severe acute diseases, and symptoms are often variable and non-specific in horses [5,12]. In general, infection with *T. equi* causes a more severe clinical disease than infection with *B. caballi* [13]. Although acute, sub-acute, and chronic forms of EP have been described, the most common condition in horses is the carrier state [12,14]. While chronically infected animals present with vague clinical signs, such as reduced exercise tolerance, carriers have extremely low or undetectable circulating parasites [15], carriers have no clinical signs [14], and act as reservoirs to the pathogen; therefore, diagnosing, controlling, and eradicating the disease can be very challenging [5,6,7]. More severe symptoms include systemic inflammatory response syndrome, increased vascular permeability, and disseminated intravascular coagulation, resulting in ischemia, anoxia, and multi-organ systemic dysfunction [6,7]. Hematological abnormalities caused by intravascular and extravascular hemolysis, microvascular stasis, and alterations in coagulation [5,6,7,12,16] are also common. Many horses become anemic, with a lower packed cell volume (PCV), hemoglobin and erythrocytes counts [5,6,7,12,17].

Serological tests are used as an indirect diagnostic method to detect antibodies raised against EP agents. The World Organization for Animal Health (OIE) recommends the use of immunofluorescence antibody tests (IFA) and complement-enzyme linked immunosorbent assays (ELISA) for diagnosing the disease [4,7,18]. However, serological assays have limited sensitivity and specificity; therefore, current diagnostic methods are based on molecular techniques, such as Polymerase Chain Reaction (PCR). PCR helps identify asymptomatic carriers, because it can detect parasitemias as low as 0.000006% and 0.017% for *B. caballi* and *T. equi*, respectively [4,6,19]. 

The global EP prevalence is consistent with the distribution of tick vectors and can vary widely among populations and geographical regions [15]. EP is endemic across almost all of Latin America, including the Caribbean countries, Central America, and South America, with the exception of the southern regions of Chile and Argentina [6,20,21,22,23,24]. The serological prevalence of *B. caballi* and *T. equi* in the Americas ranges from 5.5 to 69.2% and 14 to 88.5%, respectively [4,24,25,26,27,28,29,30], while the molecular prevalence of *B. caballi* and *T. equi* ranges from 4.4 to 60% and 24.4 to 85.7%, respectively [21,22,23,24,29]. 

Subclinical EP is present in Chile [31]; however, limited information on infection prevalence is available, even though the reporting of cases is mandatory. Most reports on piroplasmosis in Chile have relied on serosurveys, and have shown prevalence rates from 0 to 96% for *T. equi* and 0 to 2% for *B. caballi* [20,32,33,34]. Additionally, *T. equi* (19%) and *B. caballi* (1.2%) have been molecularly detected in 77 horses in the Metropolitan region [35].

The first Chilean racecourse was built in 1870, and today there are five in total, serving a population of 1600 horses. At present, there is no information regarding the circulation of *B. caballi* or *T. equi* DNA in Chilean racehorses [36]. We investigated the serological and molecular occurrence of these pathogens, and analyzed their phylogenetic and haplotype characteristics, in thoroughbred horses from racecourses in Concepción, Santiago, and Valparaíso. 

## 2. Results

### 2.1. ELISA Assessment of the Serological Occurrence of Piroplasmids

According to the ELISA results, 102 out of 286 (35.6% [95% CI: 30.6–40.6%]) horses from Chilean racecourses were seropositive. From those, 8.3% [95% CI: 5.4–12.2%] were positive for *B. caballi*, 23.7% [95% CI: 18.9–29.1%] for *T. equi*, and 3.5% [95% CI: 5.6–1.5%] for both. In Santiago and Valparaíso, 19 out of 150 (12.6% [95% CI: 7.8–19%]) and 5 out of 136 (3.6%, 95% [CI: 1.2–8.3%]) of the horses were seropositive for *B. caballi*, and 63 out of 150 (42%, 95% [CI: 34–50%]) and 5 out of 136 (3.6%, 95% [CI: 1.2–8.3%]) for *T. equi*, respectively (Table 1). 

### 2.2. Nested PCR Assay Assessment of the Molecular Occurrence of Piroplasmids 

All 457 tested DNA samples (mean (×) and standard deviation (SD) of DNA concentration = 26.5 ± 16.5 ng/µL; mean and SD 260/280 ratio = 1.87 ± 0.4) were positive for the ACTB reference gene, and there was no amplification of negative controls. According to the nPCR assays, 245 out of 457 (53.6%, 95% [CI: 49.6–56.6%]) horses were positive for piroplasmids. Of these, 202 out of 457 (44.2%, 95% [CI: 39.5–48.8%]) were positive for *T. equi*, 25 out 457 (5.4%, 95% [CI: 3.5–7.9]) for *B. caballi*, and 18 out of 457 (3.9%, 95% [CI: 2.3–6.1%]) were positive for both EP agents (*T. equi* + *B. caballi*). The nPCR analysis showed that in Concepción, Santiago, and Valparaíso, 126 out of 167 (75.4%, 95% [CI: 68.2–81.7%]), 32 out of 151 (21.1%, 95% [CI: 15.1–27.1%]), and 44 out of 139 (31.6%, 95% [CI: 24.0–40.1%]) of the horses were positive for *T. equi*, respectively; 21 out of 167 (12.5%, 95% [CI: 7.9–18.6%]), 0 out 151 (0%), and 4 out 139 (2.8%, 95% [0.8–7.2%]) were positive for *B. caballi*, respectively; and 17 out of 167 (10.1%, 95% [6.0–15.8%]), 0 out 151 (0%), and 1 out of 139 (0.7%, 95% [CI: 0.02–3.9%]) were positive for both agents, respectively (Table 1). 

### 2.3. Phylogenetic and Haplotype Analyses 

Out of the 202 positive samples for *T. equi* that were tested by targeting the complete 18S rRNA gene (1600 bp) [37], 10 amplicons that had good band quality in agarose gel were selected and submitted for sequencing (gene bank accession numbers: MT463604-MT463613) and maximum likelihood analyses (ML). These sequences, when analyzed by BLASTn (using default parameters), showed a shared identity ranging from 99% to 100% with *T. equi* sequences previously detected from equids in Israel (MN611352.1), Brazil (MG052898.1), Turkey (MG569905.1), and the Philippines (KX900447.1). Among the 25 positive samples for *B. caballi*, only one sample (MT521699) could be sequenced using the 48 KDa rhoptry protein gene protocol [38], and submitted for Bayesian analyses. The BLASTn analyses (using default parameters) revealed that this sequence shared 99% to 100% identity with *B. caballi* amplified from equine blood samples from the Philippines (KX900447.1), Egypt (KR811095.1), Mongolia (AB703053.1), and Cuba (KY111765.2). All sequences amplified in the present study showed query coverage ranging from 99% to 100%, and an E-value of 0.

**Figure 1:**
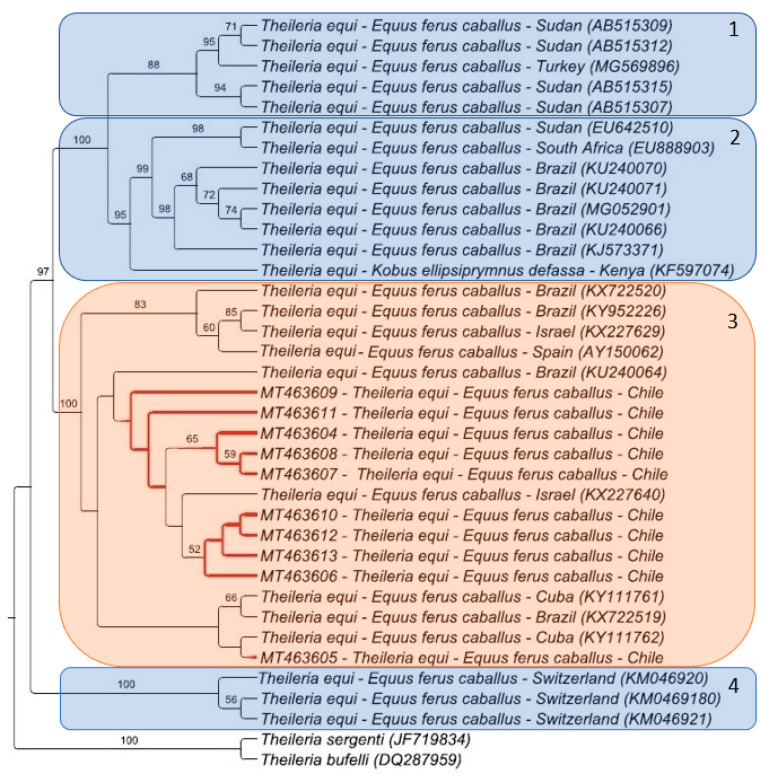
Phylogenetic tree of *Theileria equi* 18S rRNA (1600 bp) sequences. The analysis was performed using the maximum likelihood method with the TPM3uf+I substitution model. Numbers in the branches correspond to bootstrap values after 1000 repetitions. 18S rRNA sequences of *T. sergenti* and *T. bufelli* were used as outgroups. Red branches identify the sequences from this study.

**Figure 2:**
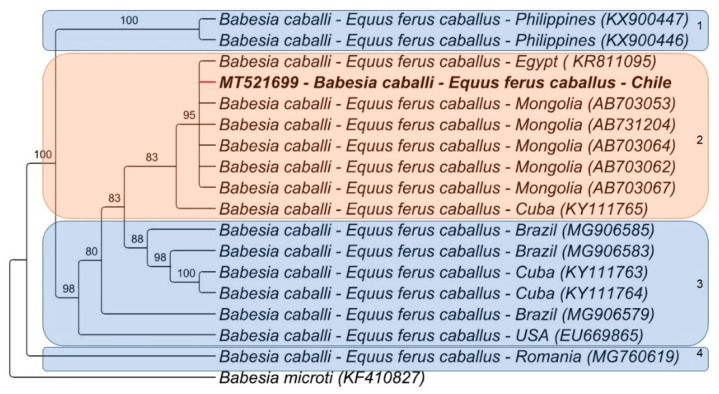
Phylogenetic tree of *B. caballi* 48 KDa rhoptry protein (430 bp) sequences. The analysis was performed using the Bayesian method with a TPM2uf substitution model. Numbers in the branches correspond to bootstrap values after 1000 repetitions. The 48 KDa rhoptry protein gene sequence of *B. microti* was used as an outgroup. The red branch indicates the sequence from this study.

While the maximum likelihood analysis of *T. equi* showed four clusters in the phylogenetic tree, the *T. equi* sequences (*n* = 10) detected in this study were all positioned in cluster #3 (Figure 1). The Chilean sequences formed a cluster with each other and with sequences found in Israel, Brazil, and Cuba (Figure 1). Bayesian analysis of *B. caballi* formed six clusters, and the sequence detected in this study was closely positioned to sequences previously detected in horses from Egypt and Mongolia (cluster #2) (Figure 2). 

**Figure 3:**
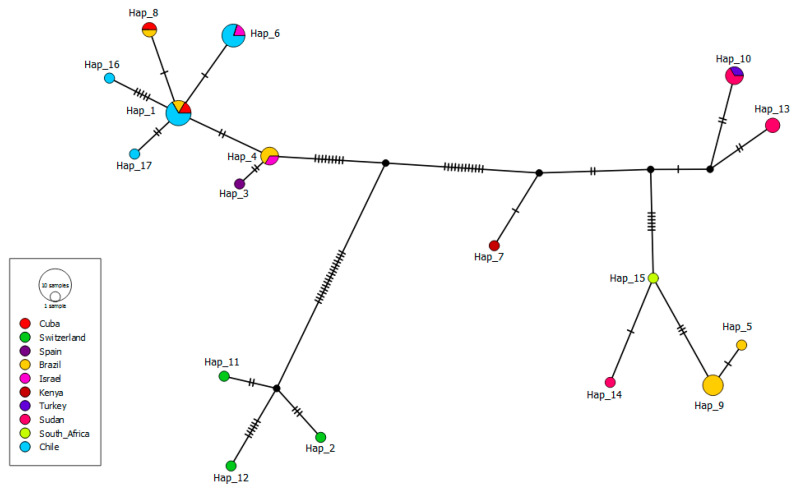
Haplotype TCS network [39] for *Theileria equi* 18S rRNA sequences (1600 bp) detected in blood samples from Chilean thoroughbred horses and others in GenBank. Each small dash indicates a mutational event. Black circles represent median vectors.

Out of 35 *T. equi* 18S rRNA sequences used for the haplotype analysis (10 from horses in this study and 25 from horses worldwide), 17 different haplotypes were detected, with a haplotype diversity of 0.934 ± 0.021 and a nucleotide diversity of 0.02186 ± 0.00196 (Table 2). Four haplotypes were detected in horses from this study, which originated from those previously detected in Brazil and Israel (haplotype #4), separated from them by a few mutational events. Haplotypes #6, #16, and #17 from Chile originated from the same haplotype, also from Chile (#1). Out of the four *T. equi* haplotypes detected in Chile, two were exclusive to this country, and the other two have also been previously found in Brazil, Cuba, and Israel (Figure 3). 

**Figure 4:**
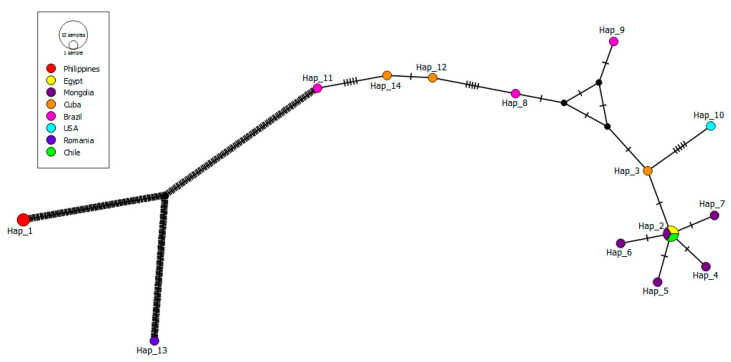
Haplotype TCS network [39] for *B. caballi* 48 KDa rhoptry protein sequences (430 bp) detected in blood samples from Chilean thoroughbred horses and others in GenBank. Each small dash indicates a mutational event. Black circles represent median vectors.

On the other hand, 17 *B. caballi* sequences (one from this study) were used for haplotype analysis, resulting in 14 different haplotypes, showing a haplotype diversity of 0.971 ± 0.032 and a nucleotide diversity of 0.21122 ± 0.082 (Table 2). The only *B. caballi* haplotype detected in Chile (#2) has also previously been found in Egypt and Mongolia (Figure 4). The haplotype from Chile originated from one previously detected in Cuba (haplotype #3), after one mutational event.

## 3. Discussion

While, in South America, *B. caballi* and *T. equi* DNA has been detected in horses from Brazil [28,29] and Venezuela [24], *B. caballi* and *T. equi* have only been molecularly reported in Chile in one study with a small sample of horses (*n* = 77) from the Metropolitan region [34]. To the best of our knowledge, this is the first molecular survey of *B. caballi* and *T. equi* in racehorses from Chile, as well the first assessment of piroplasmid haplotypes in horses from this country.

The overall molecular occurrence for EP agents obtained in thoroughbred horses from Chilean racecourses (*B. caballi* [5.4%], *T. equi* [44.2%], co-infection [3.9%]) was lower than previously described in South America (*B. caballi* [23.5%], *T. equi* [84.3%], co-infection [22%]) [40,41]. As a global trend [9], *T. equi* is more prevalent than *B. caballi*, whereas co-infection is less frequent. The higher prevalence of *T. equi* when compared to *B. caballi* is in accordance with the findings of other authors [42,43,44], which can be attributed to the lifelong persistence of *T. equi* infection, compared with the effective elimination of *B. caballi* [6,45].

Interestingly, the Club Hípico de Concepción had a higher molecular prevalence of these agents (*B. caballi* [12.5%], *T. equi* [75.4%], co-infection [10.1%]) than the other two Chilean racecourses. This racecourse showed a similar occurrence of EP to that found in research in Cuba (*B. caballi* [25%], *T. equi* [73%], co-infection [20%]) (Díaz-Sánchez et al., 2018), Mongolia (*B. caballi* [1%], *T. equi* [92.7%], co-infection [1.2%]) [46], and Spain (*B. caballi* [29.4%], *T. equi* [66%], co-infection [22.6%]) [47]. The higher occurrence of equine piroplasmids in the Club Hípico de Concepción might be related to an infection cluster caused by the management of horses (favoring transmission) and their origin (favoring the introduction of asymptomatic carriers), resulting in high numbers of asymptomatic animals [48]. It is recommended that this racecourse investigate its management of horses, the presence of ectoparasites, contaminated fomites, and environmental conditions.

The overall seroprevalence of *B. caballi* (8.4%), *T. equi* (23.7%), and co-exposure (3.5%) found in this research was higher than that in a previous study from Chilean racecourses (*B. caballi* [0%], *T. equi* [1.2–43.9%], co-infection [0%]) [49], but lower than those described in Pakistan (*B. caballi* [21.6%] and *T. equi* [41.2%]) [50], Spain (*B. caballi* [11.4%) and *T. equi* [50.3%]) [51], Italy (*B. caballi* [12.4%] and *T. equi* [17.9%]) [52], France (*B. caballi* [12.9%] and *T. equi* [50.0%]) [53], Trinidad and Tobago (*B. caballi* [68.8%] and *T. equi* [33.3%]) [25], Brazil (*B. caballi* [69.2%] and *T. equi* [78.3%]) [54], and Costa Rica (*B. caballi* [69.9%] and *T. equi* (88.5%]). 

Variations in worldwide prevalences are associated with infection status, sampling differences, the distribution of vectors, and the tests used to evaluate infection (serological methods vs. conventional PCR vs. real-time PCR) [23,55,56,57]. Moreover, worldwide serological prevalence might underestimate a novel species of *Theileria*, detected in horses from the USA. The recently described piroplasmid *T. haneyi* [58] is undetectable using current surveillance tools that target EMA-1 of the immunodominant EMA superfamily, such as the ELISA used in our study. Here, we used EMA-1 ELISA; however, previous serosurveys in thoroughbred horses from Chile have been based on Agar Gel Immunodiffusion Assay (AGID), complement fixation test (CFT), and indirect immunofluorescence antibody test (IFA) [20,32,33,35], which make specific comparisons between prevalence rates difficult. 

We observed a higher seroprevalence at the Club Hípico de Santiago racecourse (*B. caballi* [12.6%] and *T. equi* [42.0%] and co-exposure [6.0%]) compared to the Valparaíso Sporting Club (*B. caballi* [3.6%] and *T. equi* [3.7%] and co-exposure [0.7%]); however, PCR testing showed higher prevalences of infection in Valparaíso Sporting Club. Higher exposure to piroplasmids detected by the ELISA at the Club Hípico de Santiago racecourse could be due to the higher density of horses in that racetrack when compared to Valparaiso [59,60]; however, this finding might also reflect the different stages of the disease (infection vs. exposure), measured via different tests (namely, PCR and ELISA) [23,49,55,56].

We did not observe any relationship between serology and positive PCRs, reflecting the different stages of the EP disease and the characteristics of each methodology. PCR is the best technique for diagnosing animals during acute infections, when there are no detectable levels of antibodies. It is sensitive to the detection of the DNA of the parasite in early stages of the infection, being also able to detect carrier animals [9]. On the other hand, ELISA will detect antibodies, and is therefore a good test for chronic infections [7,9,23,28,55,56].

Preliminary studies of the 18S rRNA gene allowed for the detection of five *T. equi* groups, named A to E [9,61], of which four were detected in this work [9,37]. All *T. equi* samples from Chile belonged to a single group based on 18S rRNA gene sequences, and were assigned to Group A from Bhoora et al. (2009) [37]. In contrast, previous studies have shown higher genetic diversity within 18S rRNA sequences from *T. equi* in horses from Brazil [29], Jordan [62], South Africa [37], and Switzerland. A low diversity within *T. equi* sequences from Chile could be related to the history of horses’ arrival in the country, and the equid business movement trends [63]. Consistent with the above, although Chile has one of the most prestigious racecourses in Latin America [60], it is a minor participant in the global equine industry, and engages in less movement of horses [64], which in turn might have contributed to the low *T. equi* genetic diversity observed in the present study. Moreover, although Ixodidae ticks are present in Chile [65], we did not find ticks parasitizing the horses sampled in this study. 

The *B. caballi* phylogenetic analysis divided the sequences into four genotypes, in agreement with previous studies [55,66]. Even though the Chilean sequence belonged to the same group as sequences from Cuba, Egypt, and Mongolia, further studies are needed to determine the genetic variations between *B. caballi* isolates from Chile.

Herein, both *T. equi* and *B. caballi* haplotypes presented genetic proximity with sequences previously detected in other countries. Interestingly, the sequences detected in Chile might have originated from those reported in Brazil, Israel, or Cuba. Indeed, they were separated from each other by a few mutational events, which might indicate a close epidemiological link among isolates [67]. These findings also pointed out that the genetic diversity of equine piroplasmids is not geographically delimited [55]. Finally, the haplotype analysis of our sequences with others around the world showed a high level of genetic diversity in *B. caballi* (Hd: 0.971 ± 0.032) and *T. equi* (Hd: 0.934 ± 0.02) haplotypes, which have been observed worldwide [7,68,69]. This can be explained by the biphasic modes of piroplasmid reproduction (sexual and asexual), and the intensity of transmission, influenced principally in our country by mechanical or iatrogenic transmission via contaminated needles and syringes, blood transfusion, and surgical instruments [9]. Furthermore, comparison of 18S rDNA sequences classified as *T. equi* from multiple locations worldwide indicates greater genetic diversity than expected within a single species. This suggests that multiple taxa are likely to be involved, including additional, so far undescribed and novel species, as was the case for *T. haneyi* [58].

## 4. Materials and Methods

### 4.1. Animals and Areas of Study

This study was approved by the Universidad Austral de Chile (UACh) bioethics committee under UACh 238/2015. To accurately determine the occurrence of *B. caballi* and *T. equi* at the Concepción, Santiago, and Valparaíso racecourses, the required sample size was estimated according to Thrusfield (2007) [70]. The estimated prevalence was 10%, based on a preliminary study in Chile carried out by Vargas et al. (2004), with a precision of 5% and a 95% confidence interval. The minimal sample required was 139 horses from each racecourse. Each owner signed a consent form before the horses’ blood samples were taken. 

**Figure 5:**
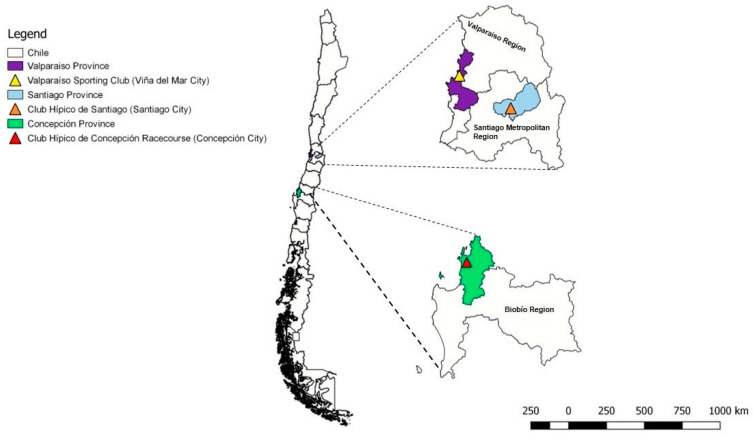
The political division of Chile, showing administrative regions where the horses were sampled.

Over 12 months (February 2015 to February 2016), 457 thoroughbred horses had their blood sampled by a veterinary team. One hundred and fifty-one horses came from the “Club Hípico de Santiago” racecourse (Santiago City, Central Chile: 33°27′00″ S, 70°40′00″ W), 139 horses from the “Valparaíso Sporting Club” (Viña del Mar City, Central Chile: 33°0′0″ S, 71°31′0″ W), and 167 from “Club Hípico de Concepción” racecourse (Concepción City, South-Central Chile: 36° 50′00″ S, 73°03′00″ W) (Figure 5). 

Blood samples (10 mL) were aseptically taken from all 457 horses via jugular venipuncture, regardless of age, gender, health, and reproductive status, and were placed into EDTA tubes for molecular analyses and serum tubes for Competitive Enzyme-Linked Immunosorbent Assay (ELISA). Samples were kept in a refrigerator box (4 °C) during transport to the UACh Veterinary Clinical Pathology Laboratory. The serum was separated, and serum and whole blood EDTA were stored at –20 °C until serological and molecular analyses were performed. 

### 4.2. ELISA for Babesia caballi and Theileria equi

A total of 150 and 136 thoroughbred horses from Santiago and Valparaíso racecourses, respectively, had their serum samples submitted for ELISA. Horses from the Concepción racecourse were not tested using ELISA as serum samples were not available. Detection of antibodies against *B. caballi* and *T. equi* by ELISA was performed using recombinant proteins RAP-1 and EMA-1, respectively, according to the manufacturer’s instructions [28,29,30]. 

OD values were read at 405 nm using an ELISA reader (Dynex Technologies, Chantilly, VA, USA). Serum samples of previously positive and negative horses were used as controls for the ELISA assays [71]. For the sera tested by ELISA, the immunological activity of each serum, the cutoff values, and the ELISA levels (EL) were calculated as described by Machado et al. (2012) [71]. The immunological activity of each serum was calculated by determining the sample to positive serum ratio (S/P), considering positive and negative sera as a reference, using the following equation: S/P = (mean sample absorbance − mean absorbance of negative serum reference)/(mean absorbance of positive reference serum − mean absorbance of negative serum reference). Cutoff values (0.334 and 0.327 for *T. equi and B. caballi*, respectively) were 2.5 times the mean absorbance value of the negative controls. The readings above the cutoff value were considered positive, according to Machado et al. [72]. 

### 4.3. DNA Extraction/Purification

DNA extraction and purification from 200 µL of each horse’s blood sample were performed using an “E.Z.N.A. Tissue DNA Kit” (E.Z.N.A. Omega BioTek^®^, Norcross, GA, USA), according to the manufacturer’s instructions, to obtain 50 µL of purified DNA. DNA concentration and purity were determined (NanoDrop ND-1000 spectrophotometer; Thermo Scientific©, Waltham, MA, USA). The absorbance ratio (OD260 nm/OD280 nm) provided an estimate of sample purity, accepting a ratio of 1.8 ± 0.2 as "pure". DNA was stored at −20 °C before performing the PCR assays.

### 4.4. Nested (n) PCR Assays 

#### 4.4.1. Endogenous Gene

A PCR for the *Equus caballus* endogenous gene beta-actin protein (ACTB) [73] was performed to check the integrity of the DNA template obtained from blood, as previously described [74] (Table 1). 

#### 4.4.2. Piroplasmids PCR DNA Controls 

The positive control for *B. caballi* was generated by gBlock® (Integrated DNA Technologies©, Coralville, IA, USA), using sequences obtained from the GenBank genetic database from different *B. caballi* RAP-1 gene sequences (KR811095.1, AB017700.1, AF092736.1, KR811097.1, EU669865.1, KR811096.1, KX900447.1, KX900446.1, AB703050.1, KY111765.2). *Theileria equi* genomic DNA was obtained from a naturally infected horses from Brazil [29]. Nuclease-free water (Thermo Scientific©, Waltham, MA, USA) was used as a negative control in all PCR reactions. 

#### 4.4.3. Nested PCR for *Babesia caballi*

All 457 samples were tested by the previously described [38] nested (n)PCR for the 48 KDa rhoptry protein gene (RAP-1), specifically for *B. caballi*. While external BC48F1 and BC48R3 primers (530 bp) were used for the first amplification reaction, internal primers BC48F11 and BC48R31 (430 bp) were used in the second reaction PCR (Table 3). The reaction mixture was composed of 12.5 μL Gotaq® Green Master Mix (Promega®, Madison, WI, USA), 200 nM of each primer (BC48F1 and BC48R3), and 5 µL of DNA template, brought to a total volume of 25 µL with nuclease-free water (Thermo Scientific©, Waltham, MA, USA). For the preliminary round of amplification, the thermal cycling protocol was as follows: initial denaturation at 94 °C for 5 min, 30 cycles of 94 °C for 30 s, 57 °C for 30 s, and 72 °C for 1 min, with a final extension at 72 °C for 5 min. The same conditions were used for the second round of amplification, except for the set of primers (BC48F11 and BC48R31), using 5 µL of the DNA product from the primary reaction as a DNA template for the nested cPCR for *B. caballi*. 

#### 4.4.4. Nested PCR for *Theileria equi*

All 457 samples were tested via the previously described nested nPCR protocol [23] to amplify a partial region of the *T. equi* 18S rRNA gene. A set of external primers (BallF and BallR (814 bp)) and internal primers (BeqF1 and BeqR1 (709 bp)) were used (Table 3). The reaction mixture was composed of 12.5 μL of Gotaq® Green Master Mix (Promega®, Madison, WI, USA), 300 nM of each primer (BallF and BallR), and 5 µL of DNA template, brought to a total volume of 25 µL with nuclease-free water (Thermo Scientific©, Waltham, MA, USA). For the primary round of amplification, the thermal cycling protocol was as follows: initial denaturation at 94 °C for 5 min, 30 cycles at 94 °C for 30 s, 57 °C for 30 s and 72 °C for 1 min, with a final extension at 72 °C for 5 min. The same conditions were used for the second round of amplification, except the set of primers (BeqF1 and BeqR1), using 5 µL of the DNA product from the primary reaction as a DNA template for the nested cPCR of *T. equi*. 

All the PCR reactions were performed in a T100 TM Thermal Cycler (Bio-Rad, Hercules, CA, USA). The PCR products were separated via electrophoresis on a 2% agarose gel (LE Agarose Seakem®, Lonza, Morristown, NJ, USA) stained with SYBR© Safe DNA gel stain (Thermo Scientific©, Waltham, MA, USA). DNA extraction/purification, cPCR amplification, and electrophoresis were performed in three separate rooms to avoid cross-contamination. The primers used in this study are shown in Table 3.

#### 4.4.5. Molecular Characterization of the *Babesia caballi* and *Theileria equi* Agents 

Selected positive *B. caballi* samples presenting strong band intensity in the RAP-1 gene were submitted for sequencing. Additionally, *B. caballi* and *T. equi* positive samples with strong band intensity were further tested by targeting the complete 18S rRNA gene (1600 bp), as previously described by Bhoora et al. (2009) [37], for sequencing and molecular characterization (Table 3). Primers NBabesia1F and 18SRev-TB were used in a primary PCR to amplify a fragment of ~1600 bp. The reaction mixture was composed of 12.5 µL Gotaq® Green Master Mix (Promega®, Madison, WI, USA), 200 nM of each primer, and 5 µL of template DNA, brought to a total volume of 25 µL with nuclease-free water (Thermo Scientific©, Waltham, MA, USA). The thermal cycling protocol was as follows: 94 °C for 2 min, followed by 40 cycles at 94 °C for 30 s, 60 °C for 45 s, and 72 °C for 1 min. Amplification was completed with a final extension of 72 °C for 7 min. 

Three nested PCRs were subsequently performed. The primers NBabesia1F and BT18S3R were used to amplify an 800 bp PCR product at the 5´-end of the gene. The amplification program was the same as that employed for the first PCR, except that an annealing step of 58 °C for 1 min was used. The amplification of the 3´-end of the 18S rRNA gene was accomplished using primers BT18S3F and 18SRev-TB, with an annealing step of 55 °C for 1 min. An internal 800 bp product, which overlapped both the 5’ and 3’ fragments by 400 bp, was amplified using primers BT18S2F and BT18S2R and an annealing step of 55 °C for 1 min.

#### 4.4.6. Sequencing, Phylogenetic, and Haplotype Analyses

The PCR products (RAP -1 and 18S rRNA genes) were purified with a commercial Silica Bead DNA Gel Extraction Kit (Thermo Scientific®, Waltham, MA, USA), following the manufacturer’s instructions, and sent to Macrogen® (Seoul, Korea) for sequencing by the Sanger method in both directions [75]. The nucleotide sequences obtained were analyzed in Bioedit v. 7.0.5.3 (Carlsbad, CA, USA), and consensus sequences were obtained from both the forward and the reverse oligonucleotides. The BLASTn analysis tool was used to compare sequences in the FASTA format with others previously deposited in the GenBank database [76]. Sequences were aligned using MAFFT software [77]. 

The alignments saved in FASTA mode were transformed into Nexus and Phylip by the Alignment Transformation Environment site, to perform Bayesian analysis using the software MrBayes 3.2.2 in the XSEDE [78] and the maximum likelihood analysis, using the cluster BlackBox RaxML BlackBox 7.6.3 [79], both via the CIPRES portal [80]. Bayesian analysis was performed with 106 generations, with varying numbers of substitution classes according to the evolutionary model found for each dataset and a burn-in = 25%. The bootstrap was accessed with 1000 replicates, and the evolutionary model was based on the Akaike Information Criterion (AIC) using the jModelTest2 software (version 2.1.6) on XSEDE [81]. Phylogenetic tree editing and rooting (via external group) were performed using the Treegraph software 2.0.56-381 beta [82].

Polymorphism nucleotide analysis of 18S rRNA and RAP-1gene sequences was performed using DnaSP v5 [83]. Haplotype diversity (Hd), number of haplotypes (*n*), and nucleotide diversity (Pi) were analyzed to explore genetic differentiation among the sampled horses. Subsequently, the sequences used in the phylogeny (from Chile and others previously deposited in GenBank) were aligned by the BioEdit Sequence Alignment Editor software [84]. The alignment was re-opened in the DnaSP version 5.10.1 software to group the sequences according to their location and saved in the NEXUS format. Then, the sequences were submitted to the analysis of the TCS network [39], which was inferred using the Population Analysis with Reticulate Trees (popART) program [85].

#### 4.4.7. Data Analysis

To determine the overall EP agent’s occurrence in racecourses from Chile, the ELISA-positive and the PCR-positive horses were divided by the total number of animals and multiplied by 100. The observed occurrence was expressed in percentages, and the 95% IC was calculated. The occurrence was also calculated per racecourse (Concepción, Santiago, and Viña del Mar). 

## 5. Conclusions

This is the first molecular and serological survey and genetic assessment of piroplasmids in Chilean Thoroughbred horses. Both *Babesia caballi* and *Theileria equi* circulate in the three leading racecourses from Chile, highlighting the impact of the pathogens on the equine industry. Our phylogenetic and haplotype analysis showed a low diversity of Chilean samples compared to the high genetic variability of piroplasmids in horses globally. The sequences we detected in Chile may have originated from Brazil, Israel, or Cuba. Further studies are needed to identify the significant vectors and routes of transmission of the pathogens in this country.

## Figures and Tables

**Table 1:** Molecular and serological occurrence of *Babesia caballi* and *Theileria equi* in Chilean Thoroughbred racehorses.

Racecourse	N	nPCR			ELISA			nPCR+ELISA Co-Positivity	
		*B. caballi* (rap-1 gene)	*T. equi * (18S rRNA gene)	Co-positivity	*B. caballi*	*T. equi*	Co-seropositivity	*B. caballi*	*T. equi*
Hipódromo de Concepción	167	12.5% (21/167)	75.4 % (126/167)	10.1 % (17/167)	NA	NA	NA	NA	NA
Club Hípico de Santiago	151	0 % (0/151)	21.1 % (32/151)	0 % (0/151)	12.6 % (19/150)	42.0 % (63/150)	6 % (9/150)	0% (0/150)	14.0% (21/150)
Valparaíso Sporting Club	139	2.8 % (4/139)	31.6 % (44/139)	0.7 % (1/139)	3.6 % (5/136)	3.7 % (5/136)	0.7 % (1/136)	0% (0/136)	2.2% (3/136)
Total	457	5.4% (25/457)	44.2.% (202/457)	3.9% (18/457)	8.4% (24/286)	23.7% (68/286)	3.5% (10/286)	0% (0/286)	8.4% (24/286)

N = Sample size; nPCR = Nested Polymerase Chain Reaction; ELISA = Enzyme-Linked Immunosorbent Assay; NA = Not Available.

**Table 2:** Polymorphism and genetic diversity of *Theileria equi* and *Babesia caballi* sequences in horses, including animals from this study.

Species	Gene	(bp)	N	VS	GC %	h	Hd (Mean ± SD)	π (Mean ± SD)	K
*Theileria equi*	18S rRNA	1100	35	61	0.45	17	0.934 ± 0.021	0.02186 ± 0.00196	17.163
*Babesia caballi*	RAP-1	350	17	289	0.5	14	0.971 ± 0.032	0.21122 ± 0.082	71.60294

N, number of sequences analyzed; VS, number of variable sites; GC, G+C content; h, number of haplotypes; hd, haplotype diversity; SD, standard deviation; π, nucleotide diversity (per site = PI); K, the average number of nucleotide differences.

**Table 3:** Summary of the different primer sets and product sizes used in conventional PCRs.

Gene	Target	Primer	Sequence (5′–3′)		Product Size (bp)	Reference
DNA Integrity checking						
Beta-actin protein	*Equus caballus*	ACTBF ACTBR	CTGGCACCACACCTTCTACA CCCTCATAGATGGGCACAGT		249	[74]
Initial Screening						
48 KDa rhoptry protein (RAP-1)	*Babesia caballi*	BC48F1 BC48R3	Primary	ACGAATTCCCACAACAGCCGTGTT ACGAATTCGTAAAGCGTGGCCATG	530	[38]
		BC48F11 BC48R31	Nested	GGGCGACGTGACTAAGACCTTATT GTTCTCAATGTCAGTAGCATCCGC	430	
18S rRNA	*Theileria equi*	BallF BallR	Primary	GTAATTCCAGCTCCAATAG AAAGTCCCTCTAAGAAGC	814	[23]
		BeqF1 BeqR1	Nested	TTCGTTGACTGCGCTTGGCG CTAAGAAGCGGAAATGAAA	709	
Molecular Characterization						
18S rRNA	Piroplasmida	NBabesia1F 18SRev-TB	Primary	AAGCCATGCATGTCTAAGTATAAGCTTTT GAATAATTCACCGGATCACTCG	1600	[37]
		NBabesia1F BT18S3R	Nested	AAGCCATGCATGTCTAAGTATAAGCTTTT CCTCTGACAGTTAAATACGAATGCCC	800	
		BT18S3F 18SRev-TB		GGGCATTCGTATTTAACTGTCAGAGG GAATAATTCACCGGATCACTCG	800	
		BT18S2F BT18S2R		GGGTTCGATTCCGGAGAGGG CCCGTGTTGAGTCAAATTAAGCCG	750	

## Data Availability

The data supporting the findings of this study are included within this article. Raw datasets generated and/or analyzed during the current study are available from the corresponding author on reasonable request. Representative sequences were submitted to the GenBank database under the accession numbers: MT463604-MT463613; MT521699.
